# Antimicrobial activities of endophytic fungi of the Sri Lankan aquatic plant *Nymphaea nouchali* and chaetoglobosin A and C, produced by the endophytic fungus *Chaetomium globosum*

**DOI:** 10.1080/21501203.2015.1136708

**Published:** 2016-02-16

**Authors:** Ranga K. Dissanayake, Pamoda B. Ratnaweera, David E. Williams, C. Dilrukshi Wijayarathne, Ravi L. C. Wijesundera, Raymond J. Andersen, E. Dilip de Silva

**Affiliations:** a Department of Chemistry, University of Colombo, Colombo, 03, Sri Lanka; b Departments of Chemistry and Earth, Ocean and Atmospheric Sciences, University of British Columbia (UBC), Vancouver, Canada; c Department of Plant Sciences, University of Colombo, Colombo, 03, Sri Lanka

**Keywords:** Endophytic fungi, *Nymphaea nouchali*, aquatic plant, *Chaetomium globosum*, antimicrobial, chaetoglobosin A, C

## Abstract

Twenty distinct endophytic fungi were isolated from the surface-sterilized plant parts of *Nymphaea nouchali* and were identified using morphological and molecular techniques. At 300 µg/disc concentration, eight of the 20 fungal extracts exhibited antimicrobial activities against *Staphylococcus aureus* (ATCC 25923) and *Bacillus cereus* (ATCC 11778) while two within the eight showed activity against *Pseudomonas aeruginosa* (ATCC 9027) and *Escherichia coli* (ATCC 35218). Furthermore, investigation of the crude extract of *Chaetomium globosum* resulted in the isolation of two known cytochalasans, chaetoglobosin A and C, and their structures were elucidated and confirmed by mass and nuclear magnetic resonance (NMR) (^1^H, ^13^C, COSY, HSQC, HMBC and tROESY) spectral data. Chaetoglobosin A showed antibacterial activities against *Bacillus subtilis* (MIC 16 µg mL^−1^), *Staphylococcus aureus* (MIC 32 µg mL^−1^) and methicillin-resistant *Staphylococcus aureus* (MRSA, MIC 32 µg mL^−1^). This is the first study to report the isolation, identification and antimicrobial properties of endophytic fungi of *N. nouchali* in Sri Lanka.

## Introduction

Endophytic fungi are ubiquitously found in the internal tissues of all plant species (Strobel and Daisy 2003). Despite the cryptic nature of their symbiotic existence in host plants they have gained recognition as prolific producers of secondary metabolites that have potential in medical and agricultural applications useful to mankind (Aly et al. 2011). However, much of the Earth’s endophytic fungal population, estimated to be in the millions, remains uninvestigated in this respect, making this an exciting frontier for useful discoveries (Cowell 2002). Only a few studies have been carried out so far to evaluate the potential of endophytic fungi inhabiting Sri Lankan biota (Ratnaweera et al. 2014, 2015a). The rich biodiversity with a relatively high level of endemism in distinct ecological settings in Sri Lanka (Myers et al. 2000; Mittermeier et al. 2005) suggests the possibility that the endophytes contained in them may have unique biosynthetic capabilities, leading to the production of unique metabolites with varied biological activities (Ratnaweera et al. 2015b).

Wetland water bodies are considered to be among the most productive ecosystems in the world with diverse forms of life including microorganisms. Aquatic plants or hydrophytes are highly adapted to their environment with unique morphological and physiological features (Yakandawala 2012) and these features are likely to make the aquatic plants unique habitats for the growth of potentially distinctive endophytic fungi. Compared to their terrestrial counterparts endophytic fungi of aquatic plants are far less investigated with respect to secondary metabolite production and their biological activities (Shearer et al. 2007; Wang et al. 2008) and more of such studies are needed to evaluate their true potential in this respect. *Nymphaea nouchali* (Nil Manel in Sinhalese) is a vulnerable aquatic plant native to Sri Lanka (MOE 2012), found in the inland freshwater bodies, rooted at the bottom, with leaves floating at the surface. The endophytic fungi of *N. nouchali* or their bioactive metabolites have not been investigated earlier. Hence, in this paper we describe the isolation and identification of the endophytic fungi of the aquatic plant *N. nouchali*, antimicrobial activities of the ethyl acetate extracts of their laboratory cultures and the bioassay-guided fractionation and structure elucidation, by nuclear magnetic resonance (NMR) spectroscopy and mass spectrometry (MS), of the bioactive constituents present in the crude extract of one of the endophytes, *Chaetomium globosum*.

## Materials and methods

### Plant material

Healthy specimens of *N. nouchali* were collected from an unpolluted natural freshwater pond in Udugampola (7.09ʹN & 79.99ʹE), in the Gampaha District, Sri Lanka, in January 2014. The identity of the plant was confirmed and authenticated by comparison with the voucher specimen in the National Herbarium at Royal Botanical Gardens, Peradeniya, Sri Lanka. A voucher specimen (No. UOC/PTS/RDNM) of the plant used for the current study was deposited in the Department of Plant Sciences, University of Colombo, Sri Lanka.

### Isolation of the endophytic fungi from N. nouchali

The collected plant specimens were brought to the laboratory in sealed polythene bags and the isolation of endophytic fungi was carried out within 5 hours of collection. First, healthy leaves, stems, sepals and petals of the plant were surface sterilized, by sequentially washing with sterile distilled water (2 min), 70% ethanol (30 sec) and 5% NaOCl solution (1.5 min). Then sterilized plant parts were surface dried with sterile filter paper and cut into 0.5 cm^2^ segments (40-leaf, 20-stem, 12-petal and 12-sepal segments) and placed on dilute Malt Yeast Agar (dMYA, HIMEDIA) enriched with the antibiotic (Cifroflaxacin 150 mg/L) in Petri dishes under aseptic conditions and incubated at room temperature for several days. The endophytic fungi that emerged from the edges of the plant segments were repeatedly subcultured on antibiotic-free sterile potato dextrose agar (PDA, Hardy-USA) dishes until pure cultures were obtained.

### Identification of fungal isolates

Initially the morphological characters (colour, colony surface, colony margin, pattern and pigment exuded) of the fungal isolates were examined. The identification of the fungal isolates was mainly performed using molecular characterization by analysing the DNA sequence of the ITS region of the ribosomal RNA gene. Each fungus sample was cultured in 100 mL of potato dextrose broth (PDB) at room temperature. Mycelium of the fungus was filtered and the isolation of genomic DNA from the mycelia of each fungal isolate was carried out according to the published protocol of Kariyawasam et al. (2012). The ITS region of the isolated genomic DNA was amplified by polymerase chain reaction (PCR) using the forward primer ITS 1 (5ʹ TCCGTAGGTGAACCTGCGG 3ʹ) and the reverse primer ITS 4 (5ʹ TCCTCCGCTTATTGATATGC 3ʹ) under the conditions, initial denaturation of 5 min at 94°C, followed by 35 cycles of 30 sec at 94°C, 1 min at 55°C and 2 min at 72°C, with a final extension of 7 min at 72°C (Diaz et al. 2012). The amplified DNA was sequenced commercially and was BLAST analysed [National Center for Biotechnology Information (NCBI)]. The acquired gene sequences were submitted to the NCBI GenBank database and accession numbers were obtained. The fungal voucher specimens were preserved on PDA slants at 4°C at the Pathology Laboratory, Department of Plant Sciences, University of Colombo, Sri Lanka.

### Fermentation, extraction and determination of antimicrobial activity

Each distinct fungal isolate was cultured on six PDA Petri dishes (120 × 20 mm) for 3–5 weeks. When the fungi were close to sporulation, each fungus together with the medium was cut into small pieces and extracted into 200 mL of ethyl acetate (EtOAc) with sonication, filtered and the filtrate was evaporated to dryness under reduced pressure (BUCHI-R-200 rotary evaporator). The resulting crude fungal extracts dissolved in methanol were tested against four pathogenic bacteria, *Staphylococcus aureus* (ATCC 25923), *Bacillus cereus* (ATCC 11778), *Pseudomonas aeruginosa* (ATCC 9027) and *Escherichia coli* (ATCC 35218) initially at 300 µg/disc, and the active extracts were further tested at 50 µg/disc concentrations using standard agar disc diffusion assay (NCCLS 2003). [+ve control – Gentamycin (10 µg/disc); -ve control – methanol] Bioassays were conducted in triplicates. After incubation overnight the mean diameter of the inhibition zones was recorded and standard error was calculated using Minitab software. The anti-fungal bioassays of crude fungal extracts were also performed in triplicate using the disc diffusion method against *Fusarium oxysporum, Rigidoporus microporus, Colletotrichum gloeosporioides and Aspergillus niger* at 300 µg/disc. Ketoconazole and Itraconazole mixture (10 µg/disc from each) was used as the positive control. The growth inhibitions were visually examined by comparing with the positive control.

Fungal strains RDNM-01, −04 and −18 showed the most promising antimicrobial activities and were selected for bioassay-guided fractionations to isolate and characterize the active compounds. This paper describes the results of RDNM-04. Research on the other two species is in progress and will be reported elsewhere. This fungal isolate (RDNM-04) was grown on sterile PDA in 250 Petri dishes (size, 120 × 120 mm) and was incubated for 18 days at 28°C until sporulation. After incubation the fungus was extracted with EtOAc (3 × 1.5 L). The extracts were filtered and the combined EtOAc extract was concentrated *in vacuo*. The crude extract was tested for antimicrobial activity against the two Gram-positive bacteria, *S. aureus* and *B. cereus,* at 50 µg/disc concentration using the agar disc diffusion method to confirm the activity. Gentamicin (10 µg/disc) was used as the positive control while methanol was used as the negative control.

### Isolation of chaetoglobosin A (1) and C (2) from Chaetomium globosum by bioassay-guided fractionation

The crude EtOAc extract of the fungal strain RDNM-04 was subjected to bioassay-guided chromatographic fractionations with reference to the antibacterial activity against *S. aureus and B. cereus*. In this process, the initial fractions obtained from column chromatography were analysed by thin layer chromatography (TLC) and fractions with similar TLC profiles were combined and the combined fractions were subjected to antibacterial bioassays by disc diffusion to detect the active fractions. First the crude EtOAc extract (2.2 g) was subjected to normal-phase silica gel chromatography (column size 2 × 25 cm) using a gradient elution from hexane, hexane/EtOAc mixtures to EtOAc to give five fractions A to E. Fractions B and C exhibited antibacterial activity. Next the active fraction B (58 mg) was fractionated by another normal phase silica column (1.5 × 35 cm) using gradient elution from hexane/EtOAc, 30:70 to EtOAc/methanol(MeOH), 30:70 to give 20 mg of an active fraction B (RDNM-04-B-B), which was finally purified by size-exclusion chromatography on Sephadex LH 20 (1.5 × 60 cm column, with MeOH:chloroform(CHCl_3_), 1:1) to obtain the active compound **2** (7.5 mg) as a white amorphous solid, which gave a single spot on TLC.

The second active fraction C (150 mg) from the first normal-phase silica chromatography was also fractionated by a normal-phase silica column (1.5 × 35 cm) using gradient elution from hexane/EtOAc, 30:70 to EtOAc/MeOH, 30:70 to give 65 mg of an active fraction B (RDNM-04-C-B). The final purification of this fraction using Sephadex LH 20 chromatography with MeOH:CHCl_3_, 1:1 led to the isolation of the pure active compound **1** (15 mg).

### Structure elucidation of 1 and 2

The structure determination of the isolated compounds **1** and **2** was performed using mass and NMR spectral data. Low-resolution electrospray ionization quadrupole ion trap MS were recorded on a Bruker-Hewlett Packard 1100 Esquire–LC system mass spectrometer while ^1^H, ^13^C and 2D NMR spectral data sets in DMSO-*d_6_* were obtained using a Bruker AV-600 spectrometer with a 5 mm CPTCI cryoprobe.

### Antimicrobial MICs of chaetoglobosin A (1) and C (2)

Antimicrobial activity of chaetoglobosin A and C was evaluated against three Gram-positive bacteria, *B. subtilis* (UBC 344), *S. aureus* (ATCC 43300) and methicillin-resistant *S. aureus* (MRSA, ATCC 33591), two Gram-negative bacteria, *E. coli* (UBC 8161) and *P. aeruginosa* (ATCC 27853), and the pathogenic fungus *C. albicans* (ATCC 90028). The minimum inhibitory concentrations (MICs) were determined using the broth micro-dilution method according to National Committee for Clinical Laboratory Standards with modification using Mueller Hinton broth as the medium (NCCLS 2002). The MIC end point was taken as the lowest concentration with more than 90% growth inhibition. Optical density of the microbial growth was determined (at 600 nm) using a DTX 880 (Beckman Coulter Inc.) plate reader. The commercial antimicrobial agents polymyxin B for *B. subtilis, E. coli* and *P. aeruginosa*, rifamycin for *S. aureus* and MRSA and amphotericin for *C. albicans* were used as positive controls (concentration series used: 2.0–0.004 µg mL^−1^).

## Results and discussion

### Isolation and identification of the endophytic fungi

Twenty distinct endophytic fungi (eight from leaves, and four each from stems, sepals and petals) were isolated in this study. Their colony morphological characteristics are listed in Table 1. Molecular characterization of the fungal isolates was performed based on the ITS sequence data and the closest BLAST match. According to the BLAST analysis, 19 fungal isolates (all except RDNM-21) showed greater than 97% identity with previously reported fungal species in NCBI Genbank and therefore their identities were established as shown in Table 1. The relevant accession numbers obtained from the NCBI Genbank for the submitted sequences are also given in Table 1.The BLAST results indicated that RDNM-21 is an *Endomelanconiopsis* sp., with only 86% homology with the reported data for KF746076 (Table 1). The 20 endophytic fungi isolated belong to 12 genera with each genus *Fusarium* and *Colletotrichum* having four species, and *Endomelanconiopsis* and *Curvularia* having three and two species, respectively, and the rest of the genera present as a single species.

**Table 1.** Identities of isolated endophytic fungi with their origin, morphological characters and accession numbers.

No	Identification code	Origin	Morphological characters					Taxon	GenBank accession no.	Closest blast match (GenBank accession No.)	Query/reference ITS length (Similarity%)
			Colour	Colony surface	Colony margin	Pattern	Pigment exuded				
1	RDNM-01	Sepals	Yellow	Cottony	Smooth	Zonate	No	*Fusarium solani*	KP326323	*Fusarium solani* (KM458800)	539/540(99%)
2	RDNM-03	Leaves	Light purple	Cottony	Restricted	Radiate	Yellowish brown	*Fusarium verticillioides*	KP419975	*Fusarium verticillioides* (KM434131)	524/525(99%)
3	RDNM-04	Leaves	Brown	Embedded	Irregular	Radiate	Yellowish brown	*Chaetomium globosum*	KP730436	*Chaetomium globosum* (KM268672)	445/460(97%)
4	RDNM-05	Stem	Dark green	Sloppy	Spreading	Irregular	No	*Trichoderma ghanense*	KP419976	*Trichoderma ghanense* (KJ807358)	560/560(100%)
5	RDNM-07	Stem	Black	Embedded	Restricted	Radiate	No	*Phyllosticta capitalensis*	KP419977	*Phyllosticta capitalensis* (KJ883595)	478/478(100%)
6	RDNM-08	Leaves	White & Pink	Cottony	Smooth	Zonate	No	*Fusarium* sp.	KP419978	*Fusarium* sp. (DQ480359)	527/528(99%)
7	RDNM-09	Stem	White	Embedded	Irregular	Radiate	No	*Phomopsis* sp.	KP684914	*Phomopsis* sp. (KM510387)	547/548(99%)
8	RDNM-11	Petals	White	Sticky & Sloppy	Irregular	Radiate	No	*Colletotrichum gloeosporioides*	KP730438	*Colletotrichum gloeosporioides* (JQ936214)	550/550(100%)
9	RDNM-13	Leaves	Black	Cottony	Spreading	Arachnoid	No	*Neofusicoccum parvum*	KP730440	*Neofusicoccum parvum* (KJ193648)	540/541(99%)
10	RDNM-14	Leaves	Grey	Sloppy	Irregular	Radiate	No	*Colletotrichum tropicale*	KP684913	*Colletotrichum tropicale* (KM357563)	499/499(100%)
11	RDNM-15	Leaves	Grey	Sloppy & Sticky	Irregular	Irregular	No	*Colletotrichum siamense*	KP684916	*Colletotrichum siamense* (KM268865)	541/544(99%)
12	RDNM-16	Stem	Dark Brown	Velvety	Smooth	Zonate	No	*Alternaria lonqipes*	KP684917	*Alternaria lonqipes* (KJ722535)	548/548(100%)
13	RDNM-17	Sepals	White & Grey	Sticky	Restricted	Radiate	Yellow	*Curvularia papendorfii*	KP684918	*Curvularia papendorfii* (KC592365)	572/572(100%)
14	RDNM-18	Leaves	Brown	Cottony	Restricted	Zonate	Light brown	*Berkleasmium* sp.	KP684919	*Berkleasmium* sp. (DQ280263)	501/503(99%)
15	RDNM-19	Stem	Black	Velvety	Smooth	Zonate	No	*Curvularia lunata*	KP730441	*Curvularia lunata* (KC288113)	574/574(100%)
16	RDNM-20	Leaves	Yellowish grey	Sloppy	Spreading	Radiate	No	*Colletotricum capsici*	KP751931	*Colletotricum capsici* (KM213014)	524/524(100%)
17	RDNM-21	Stem	Greenish grey	Sloppy	Irregular	Radiate	Yellowish brown	*Endomelanconiopsis* sp.	KP751935	*Endomelanconiopsis* sp. (KF746076)	436/506(86%)
18	RDNM-22	Sepals	Grey	Sloppy	Irregular	Radiate	Brown	*Endomelanconiopsis endophytica*	KP751932	*Endomelanconiopsis endophytica* (GQ469968)	553/559(99%)
19	RDNM-23	Leaves	Greenish grey	Sloppy	Restricted	Radiate	Yellowish brown	*Endomelanconiopsis* sp.	KP751933	*Endomelanconiopsis* sp. (KJ588255)	504/505(99%)
20	RDNM-24	Petals	Dark grey	Sloppy	Spreading	Radiate	No	*Setosphaeria rostrata*	KP751934	*Setosphaeria rostrata* (KJ439663)	573/576(99%)

These isolated fungi belong to two Ascomycota classes, Sordariomycetes and Dothideomycetes. *Fusarium, Trichoderma, Chaetomium, Colletotrichum* and *Phomopsis* species belong to the class Sordariomycetes, while *Berkleasmium, Alternaria, Curvularia, Setosphaeria, Phyllosticta, Neofusicoccum* and *Endomelanconiopsis* belong to class Dothideomycetes. *Endomelanconiopsis* sp. and Trichoderma sp. are eminent endophytic fungal groups while *Fusarium, Colletotrichum, Curvularia* and *Phomopsis* are well known as epiphytes and phytopathogenic fungi although non-pathogenic endophytic forms of these also exist (Strange and Scott 2005; Yeasmin and Shamsi 2013). Some endophytic fungi remain latent inside the host plant without producing any symptoms of disease until the environmental conditions are favourable for the fungus to turn into a pathogenic form (Sieber 2007; Rodriguez and Redman 2008). However, because all the fungi in the current investigation were isolated from healthy plant parts after thorough surface sterilization, the fungi obtained should have existed as endophytic forms during the time they have been isolated.

### Antimicrobial activity of the crude endophytic fungal extracts

The antimicrobial activities (mean diameter (± Standard error (SE)) of the inhibition zones) of the crude organic extracts of the isolated endophytic fungi are given in Table 2. Out of the 20 fungal extracts, eight (40%) were active against at least one bacterium tested while 12 (60%) were inactive against all four bacteria examined. Of the eight active extracts, all inhibited the growth of *S. aureus* and *B. cereus* at 300 µg/disc while only two extracts (RDNM-01 and RDNM-18) were active against *P. aeruginosa* and *E. coli* at this concentration. At 50 µg/disc RDNM-01, RDNM-04, RDNM-18 and RDNM-22 were active against *S. aureus* and *B. cereus* while only RDNM-1 and RDNM-18 were active against *P. aeruginosa* and *E. coli*. Two endophytic fungal extracts (RDNM-01 and RDNM-18) showed weak inhibitions against *R. microporus* and *F. oxysporum* while RDNM-22 showed weak inhibitions only against *R. microporus* at 300 µg/disc. Accordingly, out of all, *Fusarium solani* (RDNM-01), *Chaetomium globosum* (RDNM-04), *Berkleasmium* sp. (RDNM-18) and *Endomelanconiopsis* sp. (RDNM-22) can be considered as the endophytic fungi that showed promising antimicrobial activities.

**Table 2.** Antibacterial activity of the crude extracts of endophytic fungi of the plant *N. nouchali*.

Identification No. of Fungi	Origin	Antibacterial activity (Mean inhibition zone diameter ± Standard error – mm)							
		*S. aureus*		*B. cereus*		*P. aeruginosa*		*E. coli*	
		300 µg/disc	50 µg/disc	300 µg/disc	50 µg/disc	300 µg/disc	50 µg/disc	300 µg/disc	50 µg/disc
RDNM-01	Sepals	17.0 ± 0.2	12.2 ± 1.2	22.0 ± 0.3	12.2 ± 0.8	08.4 ± 0.5	07.0 ± 0.3	15.3 ± 1.4	08.1 ± 0.9
RDNM-04	Leaves	16.5 ± 1.3	13.2 ± 0.5	19.0 ± 0.6	10.1 ± 0.2	–	–	–	–
RDNM-13	Stem	10.8 ± 0.3	–	11.3 ± 0.8	–	–	–	–	–
RDNM-18	Leaves	25.0 ± 0.2	15.1 ± 1.4	27.3 ± 1.6	16.8 ± 1.1	08.0 ± 0.6	08.0 ± 0.1	20.0 ± 2.3	09.5 ± 0.3
RDNM-20	Leaves	10.0 ± 0.4	–	12.1 ± 0.1	–	–	–	–	–
RDNM-21	Stem	08.4 ± 0.2	–	08.8 ± 0.5	–	–	–	–	–
RDNM-22	Sepals	12.1 ± 0.4	07.3 ± 0.6	14.0 ± 0.9	08 ± 1.0	–	–	–	–
RDNM-23	Leaves	09.1 ± 0.2	–	07.6 ± 0.7	–	–	–	–	–
+Ve control*		23.1 ± 1.0	21.7 ± 0.9	20.6 ± 0.8	22.3 ± 2.6	19.6 ± 2.1	21.4 ± 1.8	22.4 ± 1.5	24.0 ± 1.9
-Ve control**		–	–	–	–	–	–	–	–

Notes: [* +Ve control – Gentamycin (10 µg/disc), ** – Ve control – methanol]

### Structure elucidation of the bioactive metabolite of the C. globosum

**Figure 1.**
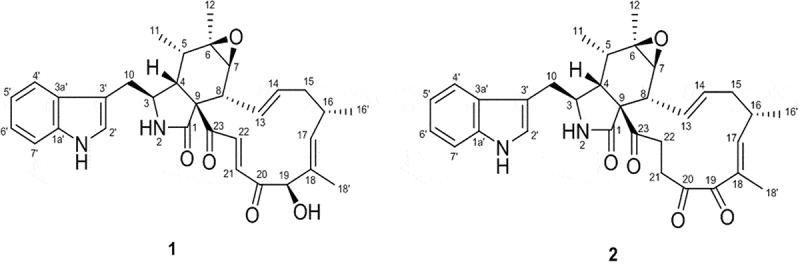
Chemical structures of chaetoglobosin A (**1**) and C (**2**).

The [M + H]^+^ ion for compound **1** and [M+Na]^+^ ion for the compound **2** gave an *m/z* 529 and 551, respectively, in their low-resolution electrospray ionization mass spectrum, consistent with a molecular weight of 528 daltons for both compounds and a molecular formula of C_32_H_36_N_2_O_5_. Analysis of ^1^H, ^13^C and 2D NMR (COSY, HSQC, HMBC, tROESY) spectral data in DMSO-*d_6_* revealed that the structures of compounds **1** and **2** match those of the known chaetoglobosin A (**1**) and C (**2**) (Figure 1), respectively. A comparison of ^13^C NMR values obtained in the present study for chaetoglobosin A (**1**) and chaetoglobosin C (**2**) with already-reported data is given in Table 3 (Sekita et al. 1983). Chaetoglobosin A and C are members of the cytochalasan family of compounds, first isolated from the same fungus *Chaetomium globosum* from Japan in 1976 together with the chaetoglobosin D, E and F.

**Table 3.** Comparison of ^13^C NMR data of chaetoglobosin A and C from the present study (in DMSO-*d_6_*) with published data (in DMSO-*d_6_*) (Sekita et al. 1983).

	1		2		^13^C δ (ppm)	1		2	
C#	Reported values	Present values	Reported values	Present values	C#	Reported values	Present values	Reported values	Present values
1	172.5	172.7	173.8	173.9	17	137.5	137.5	155.6	155.9
2	–	–	–	–	18	131.5	133.5	130.9	131.1
3	52.1	45.9	52.2	52.4	18’	10.5	10.6	9.9	10.1
4	45.9	40.1	48.4	48.5	19	81.5	81.6	208.0	208.3
5	35.3	35.2	36.1	36.2	20	200.3	200.4	205.2	205.5
6	57.0	52.2	56.9	56.8	21	135.3	131.6	31.8	31.9
7	61.3	57.0	60.3	60.3	22	133.7	136.0	37.0	37.2
8	46.0	45.8	48.3	48.2	23	199.2	199.5	196.0	196.3
9	62.9	61.2	62.3	62.4	2’	120.7	124.0	120.8	121.0
10	32.6	32.7	31.9	31.8	3’	109.1	109.2	108.0	108.0
11	12.1	12.2	12.3	12.4	3a	127.2	127.1	127.6	127.8
12	19.0	19.1	18.9	19.1	4’	117.9	120.8	118.2	118.4
13	127.2	127.4	127.0	127.0	5’	123.9	118.5	125.0	125.6
14	133.2	133.7	133.1	133.1	6’	118.3	118.1	118.2	118.8
15	40.2	40.0	39.5	39.1	7’	111.3	111.4	111.2	111.4
16	31.7	31.8	32.5	32.6	1a’	136.0	136.1	135.8	135.9
16’	20.8	20.9	19.3	19.4

### Antimicrobial activity of chaetoglobosin A (1) and C (2)

In the present investigation chaetoglobosin A (**1**) showed good antibacterial activities against Gram-positive *B. subtilits* (UBC 344), MRSA (ATCC 33591) and *S. aureus* (ATCC 43300) with MIC values of 16, 32 and 32 µg mL^−1^, respectively. The MIC for the positive controls, polymixin B, is 8 µg mL^−1^ for *B. subtilis* and 0.015 µg mL^−1^ for rifamycin against *S. aureus* and MRSA. The MIC values for chaetoglobosin C were >64 µg mL^−1^ for all the microorganisms tested. This is the first report of antimicrobial activities of chaetoglobosin A and C.

## Conclusion

This is the first study to isolate the endophytic fungi of the native aquatic plant *N. nouchali* and investigation of their antimicrobial activities. This study showed that the aquatic plant *N. nouchali* harbours many endophytic fungi that are capable of producing antimicrobial substances. Although the chaetoglobosin A and chaetoglobosin C isolated from the fungal extract RDNM-04 proved to be known compounds, the possibility of isolating new biologically active compounds from other active endophytic fungal extracts still remains. Thus it can be concluded that the endophytic fungi of *N. nouchali* are a valuable potential source for the isolation of bioactive metabolites and more investigations should be performed to realize its true potential.

## Supplementary Material

Supplementary_material.pdf
