# Supplementary material for: Antimicrobial activities of endophytic fungi of the Sri Lankan aquatic plant Nymphaea nouchali and chaetoglobosin A and C, produced by the endophytic fungus Chaetomium globosum
Source: Mycology. 2016 Feb 16;7(1):1–8. doi: 10.1080/21501203.2015.1136708 (PMC6059152; doi:10.1080/21501203.2015.1136708)
Supplement: Supplementary_material.pdf [file TMYC_A_1136708_SM5423.pdf]

Supporting Documents

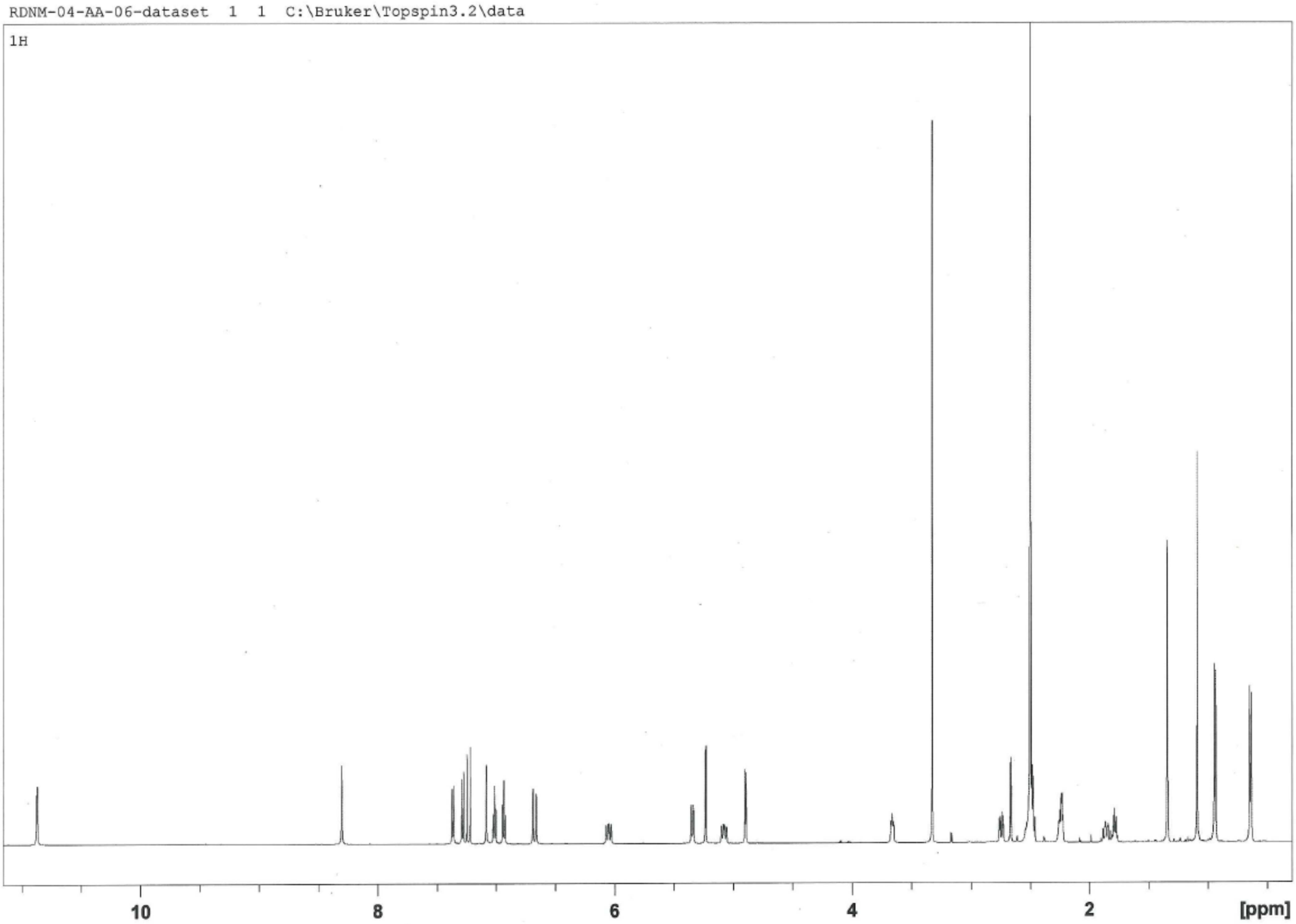

The ^{1}H NMR spectrum (600 MHz) of Chaetoglobosin A (**1**), recorded in DMSO-d_6

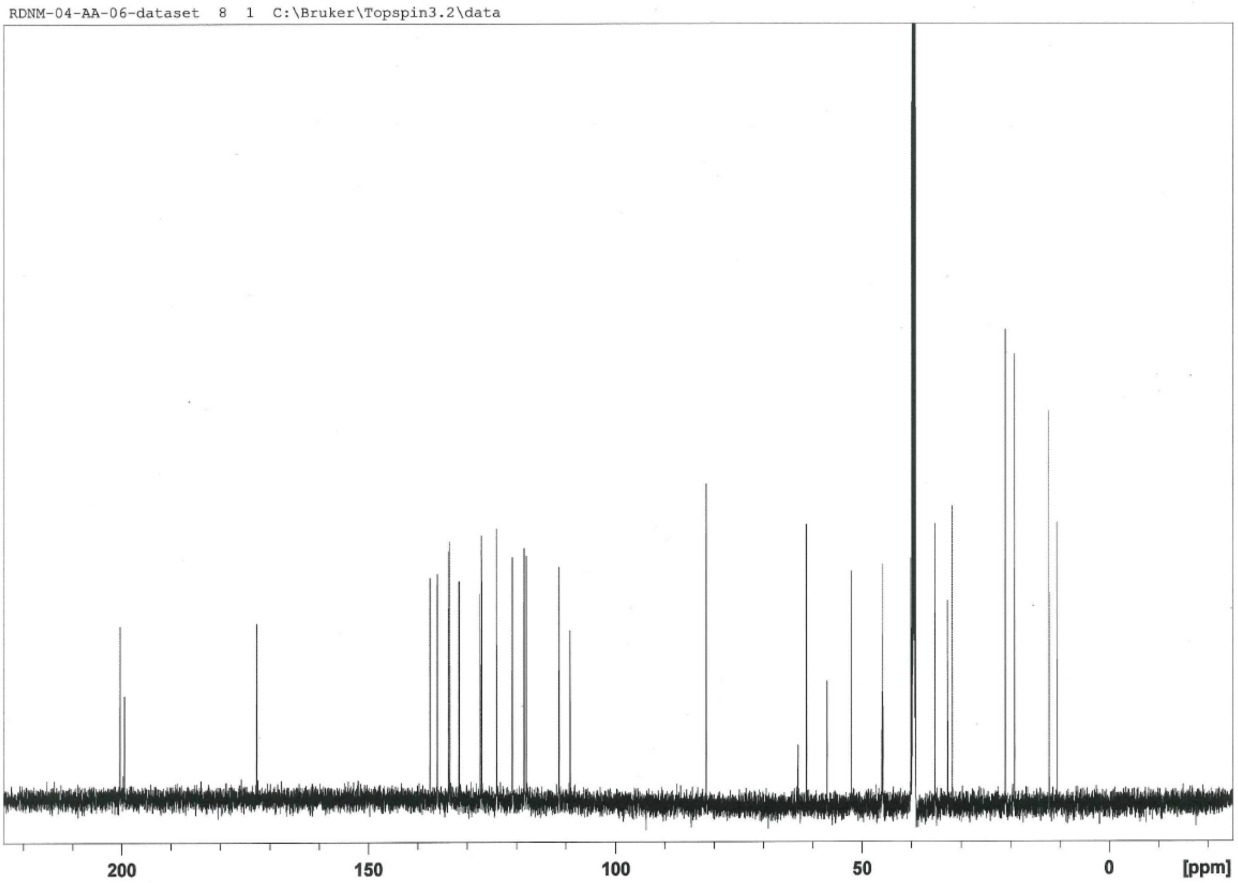

The ^{13}C NMR spectrum (150 MHz) of Chaetoglobosin A (**1**), recorded in DMSO-d_6

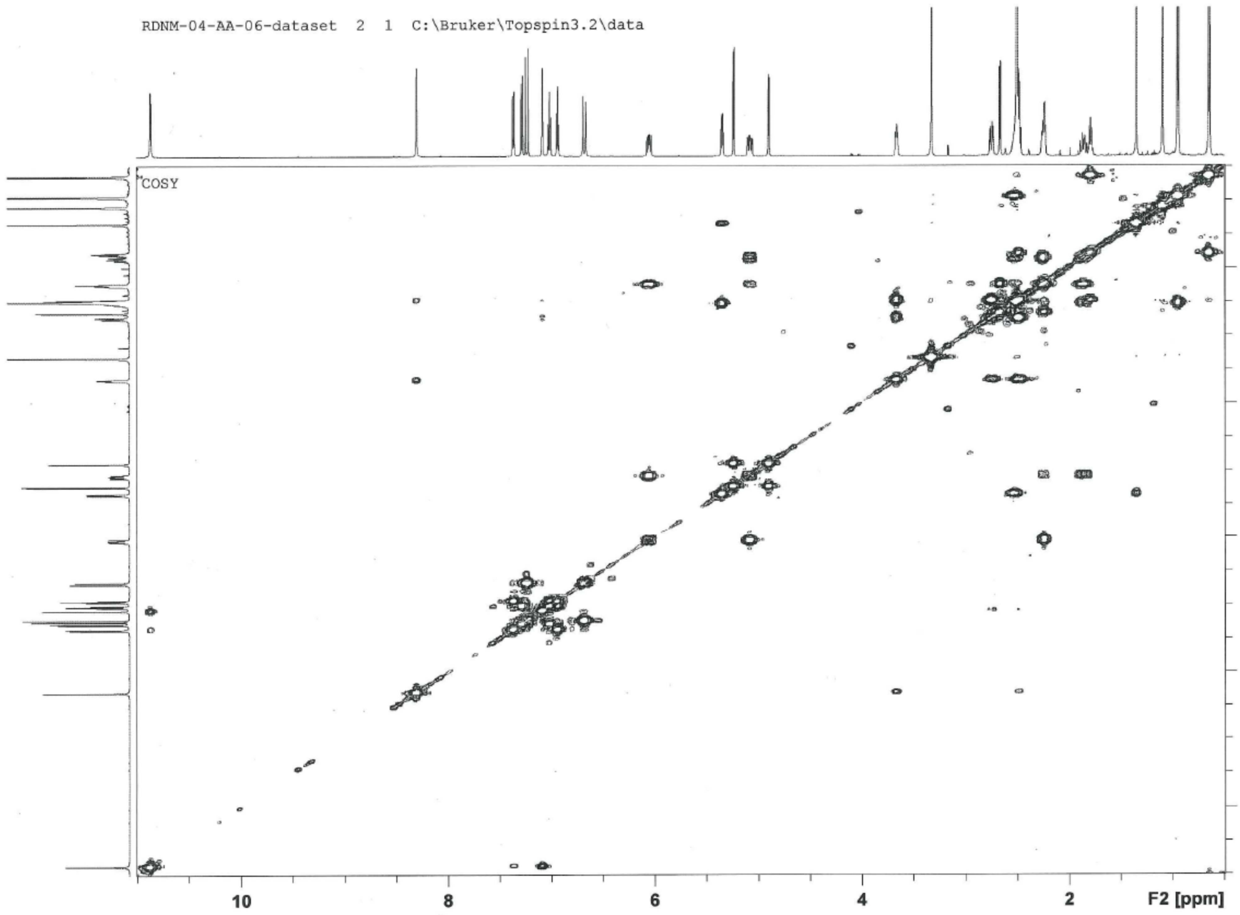

The COSY NMR spectrum (600 MHz) of Chaetoglobosin A (**1**), recorded in DMSO-d_6

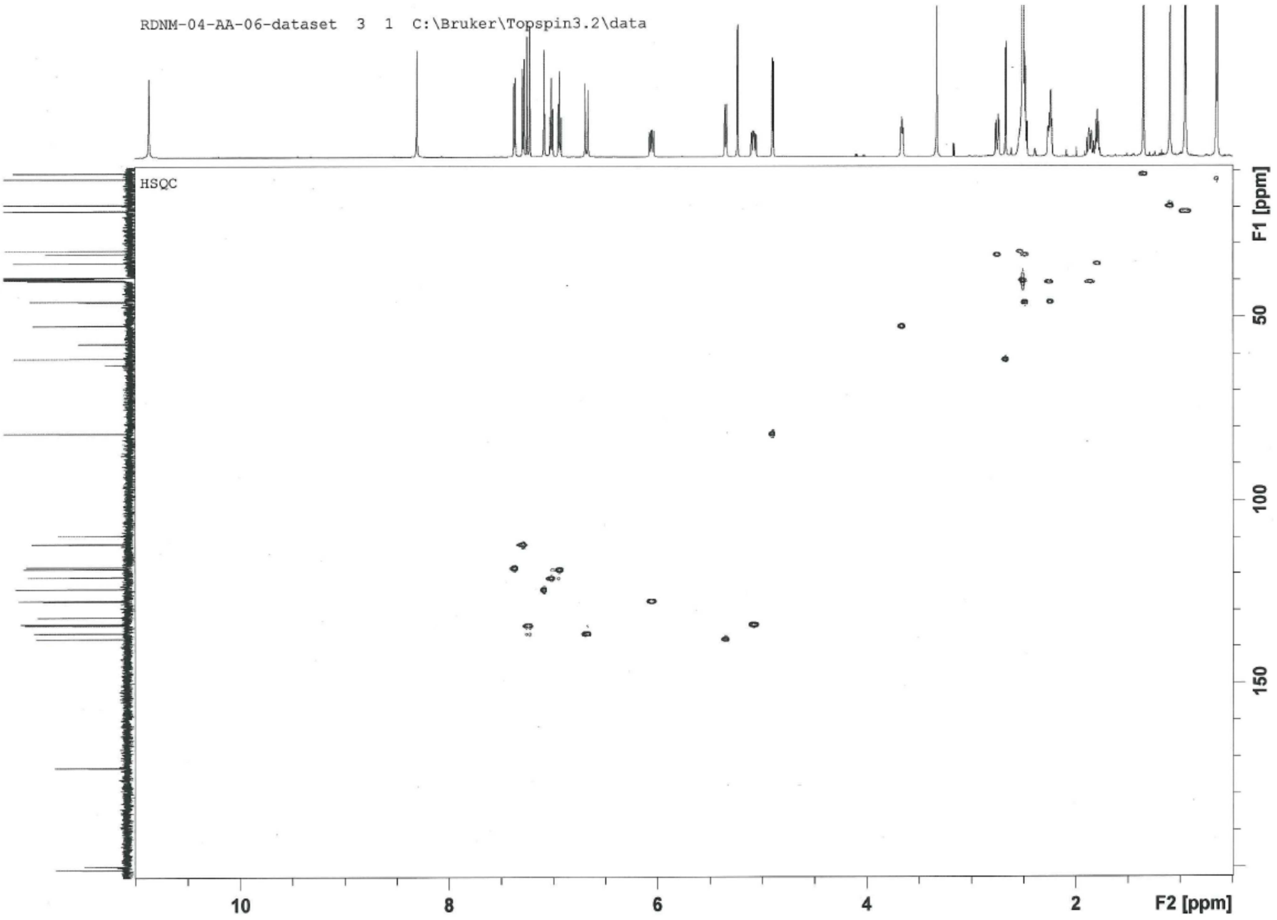

The HSQC NMR spectrum (600 MHz) of Chaetoglobosin A (**1**), recorded in DMSO-d_6

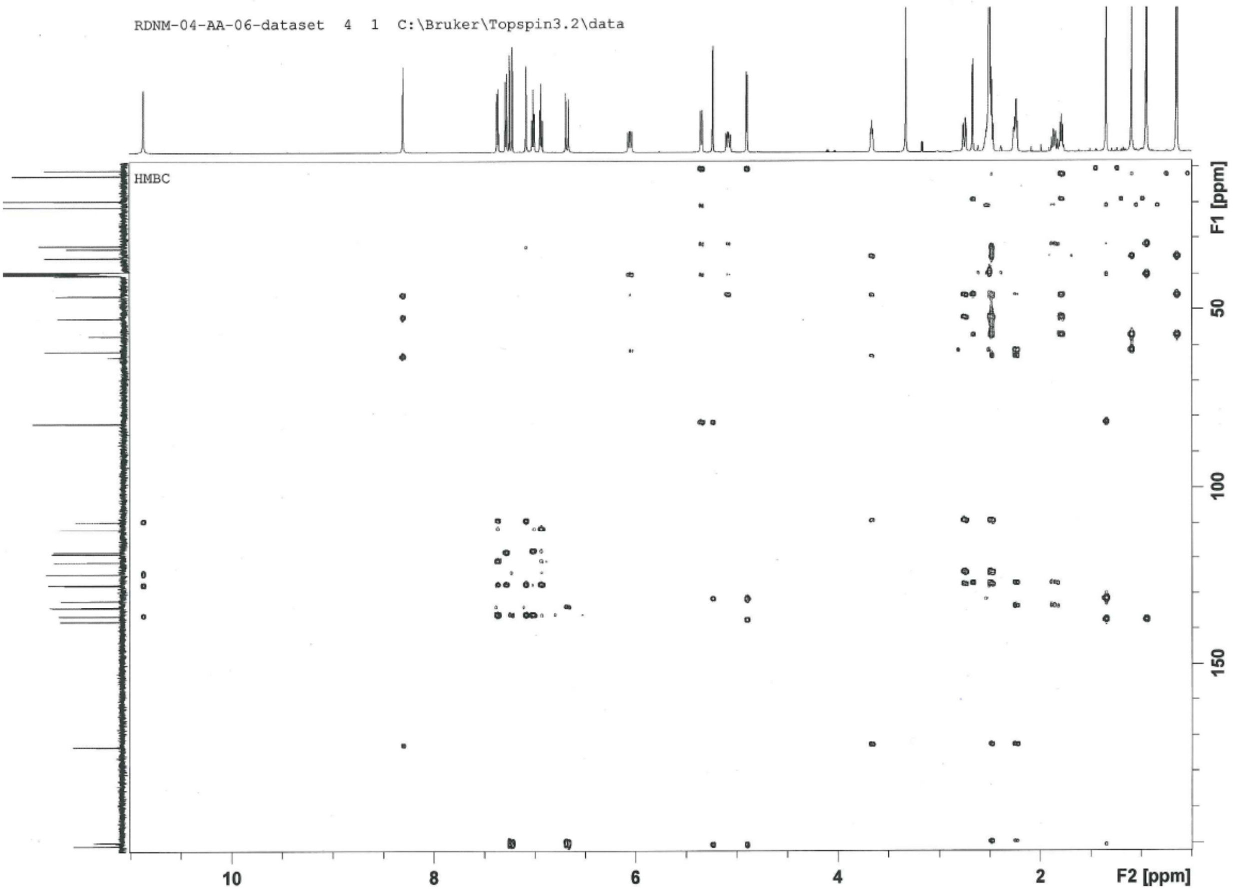

The HMBC NMR spectrum (600 MHz) of Chaetoglobosin A (**1**), recorded in DMSO-d_6

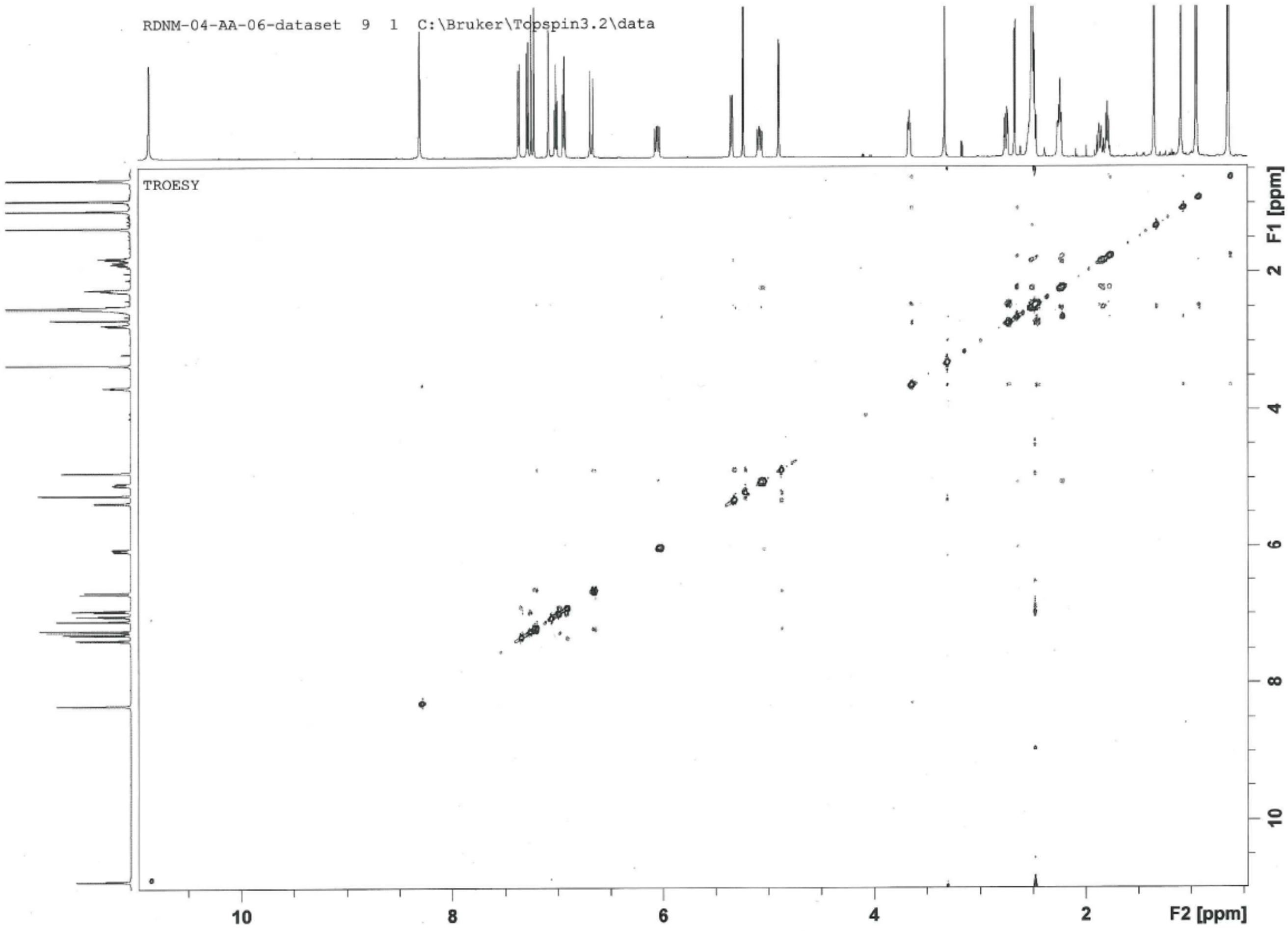

The tROESY NMR spectrum (600 MHz) of Chaetoglobosin A (**1**), recorded in DMSO-d_6

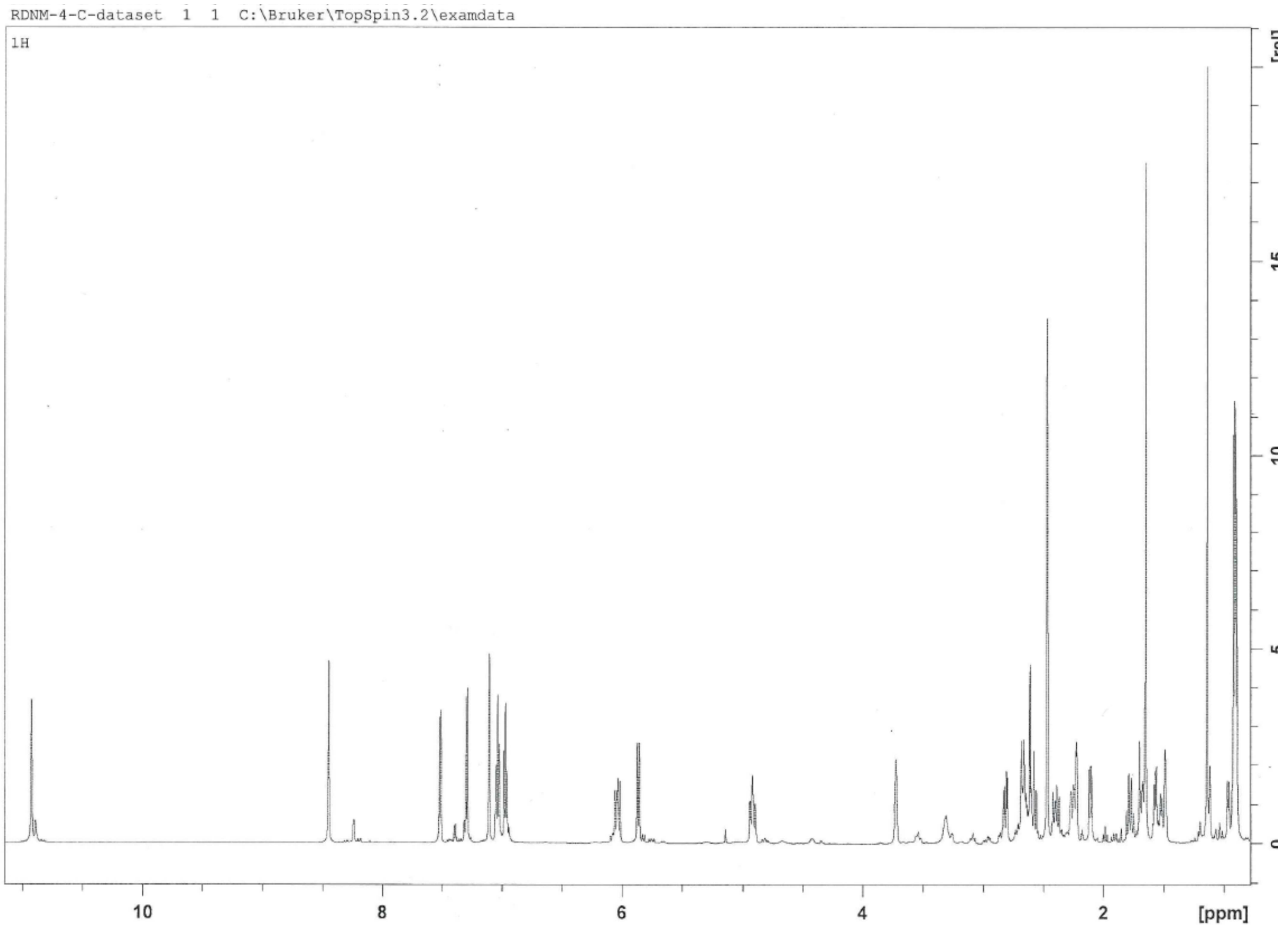

The ^{1}H NMR spectrum (600 MHz) of Chaetoglobosin C (**2**), recorded in DMSO-d_6

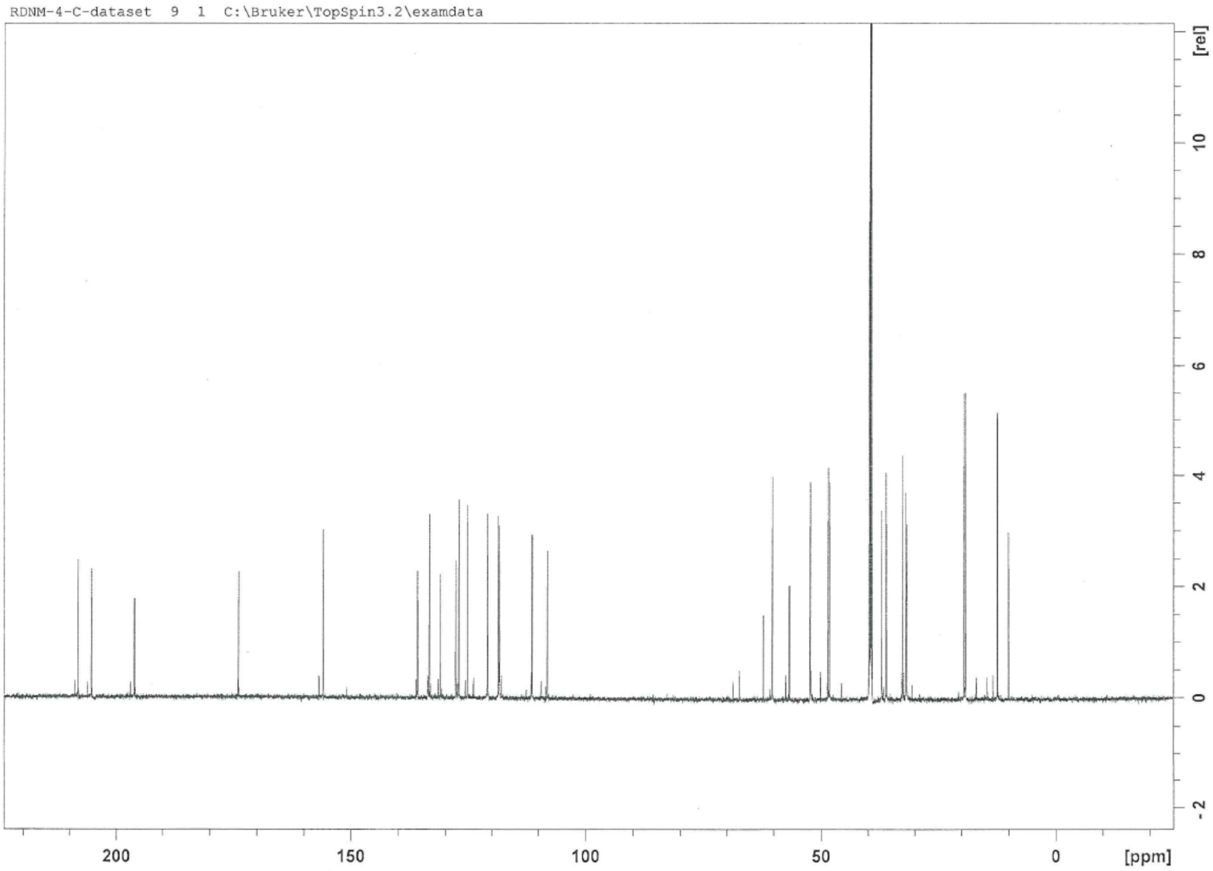

The ^{13}C NMR spectrum (150 MHz) of Chaetoglobosin C (**2**), recorded in DMSO-d_6

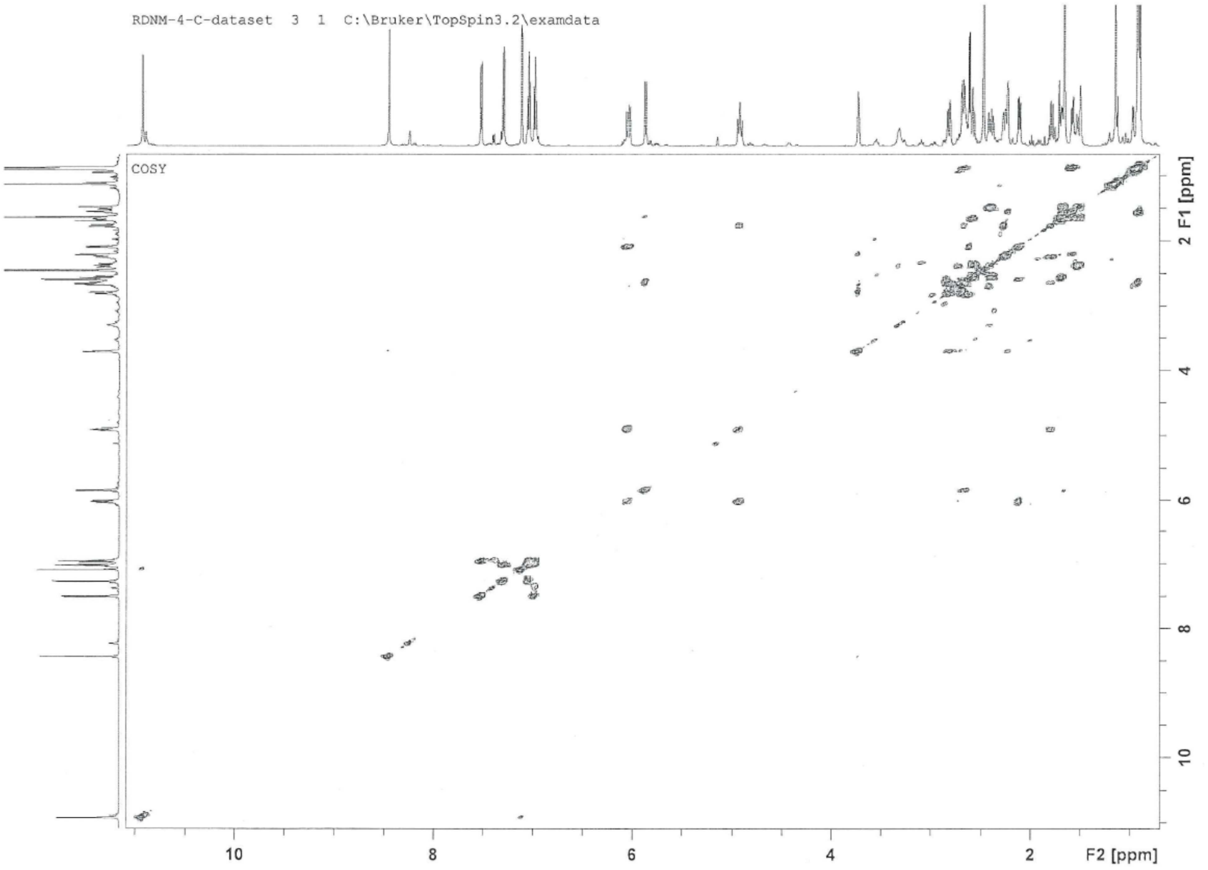

The COSY NMR spectrum (600 MHz) of Chaetoglobosin C (2), recorded in DMSO-d_6

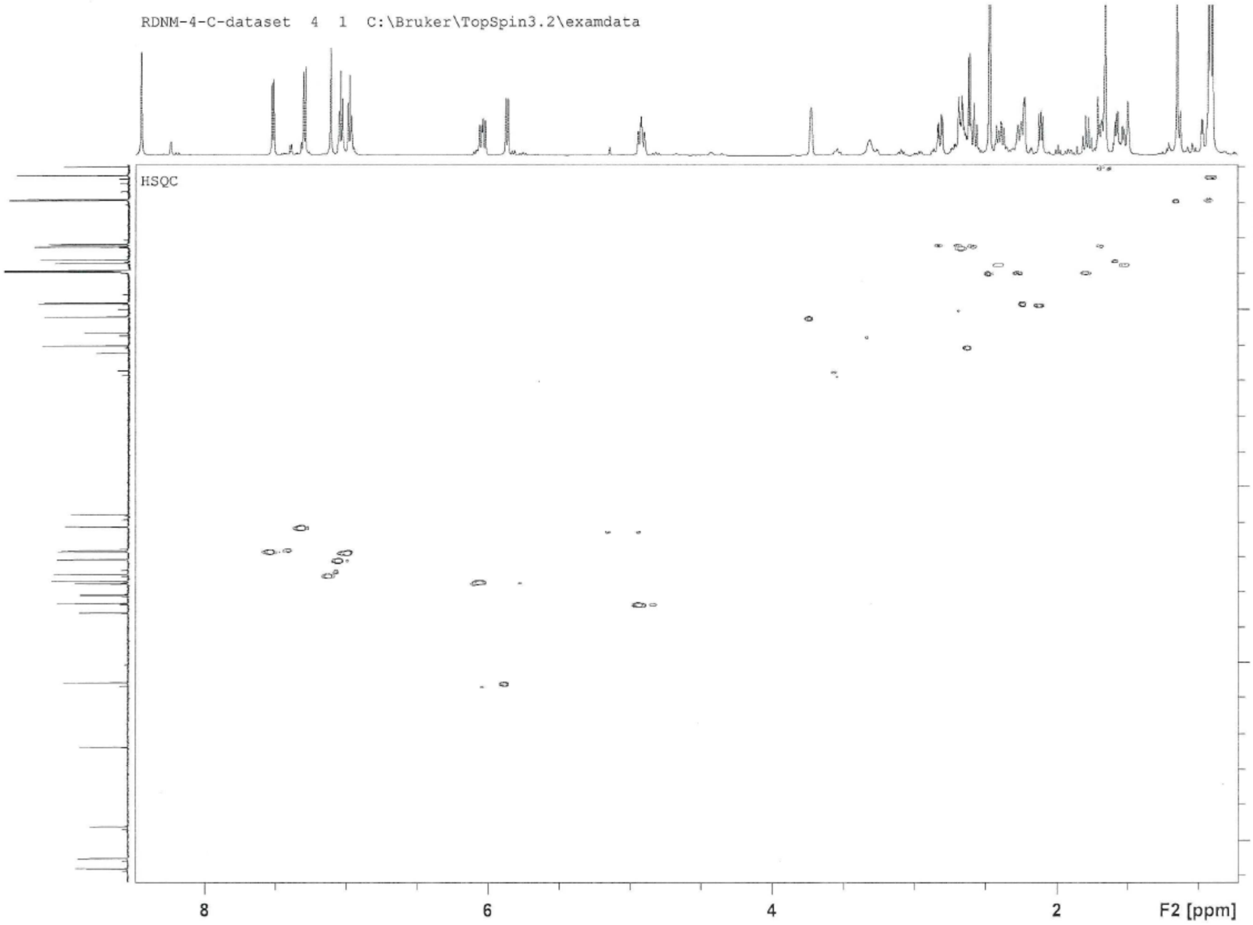

The HSQC NMR spectrum (600 MHz) of Chaetoglobosin C (**2**), recorded in DMSO-d_6

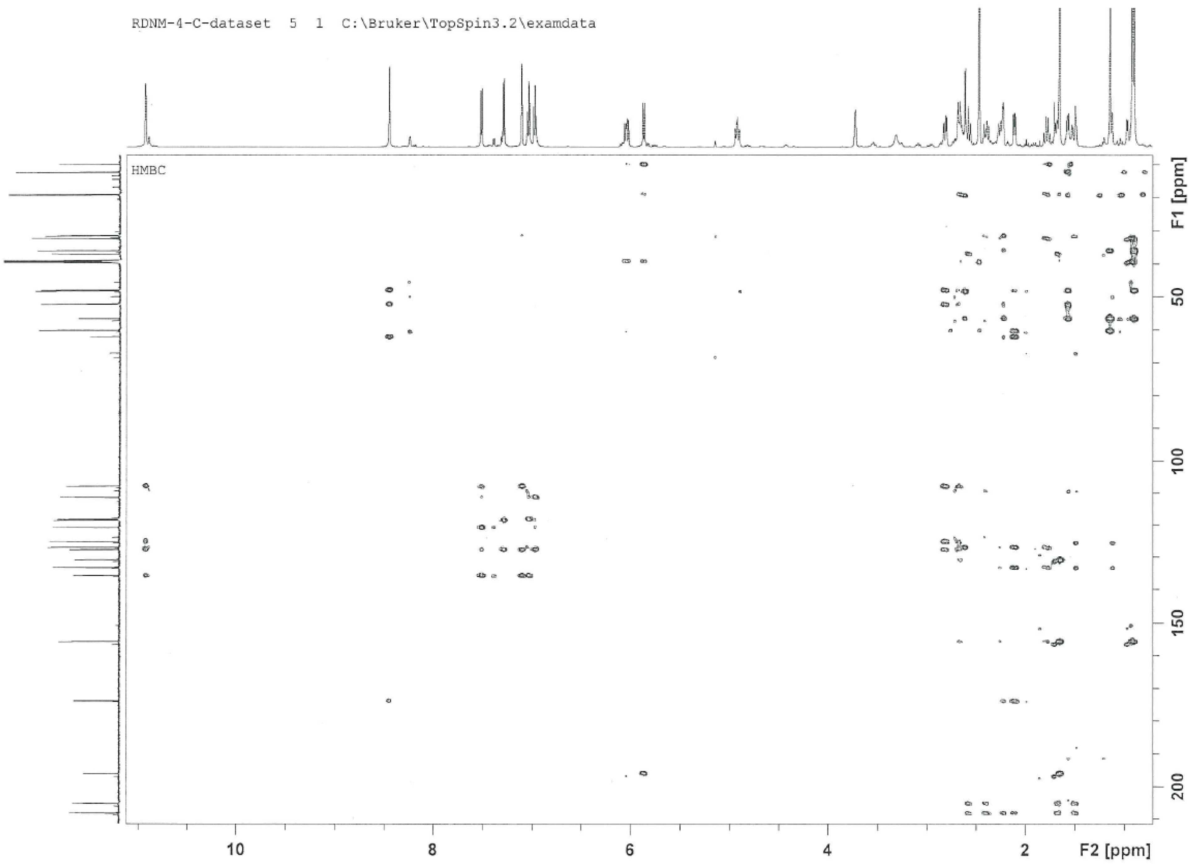

The HMBC NMR spectrum (600 MHz) of Chaetoglobosin C (**2**), recorded in DMSO-d_6

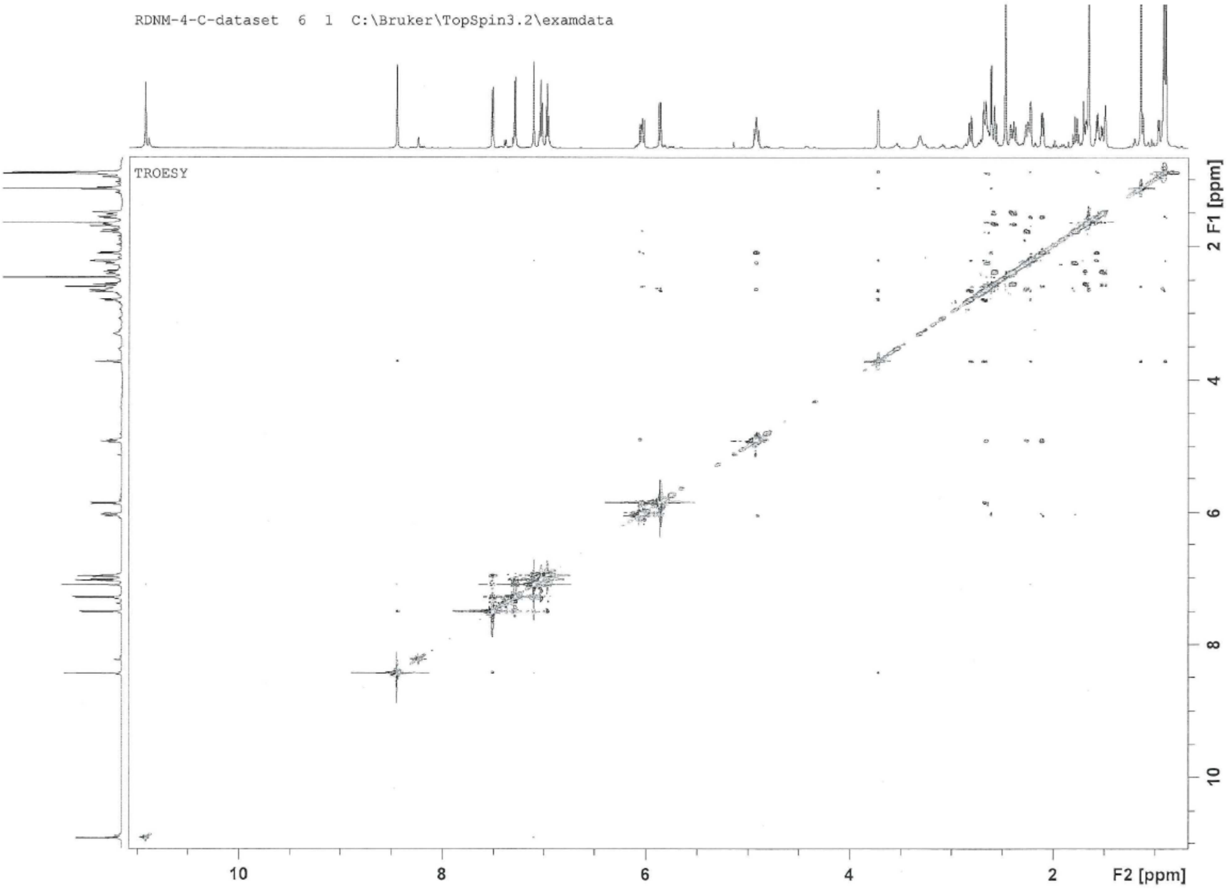

The tROESY NMR spectrum (600 MHz) of Chaetoglobosin C (**2**), recorded in DMSO-d_6
